# Association between preeclampsia in daughters and risk of cardiovascular disease in parents

**DOI:** 10.1007/s10654-023-00972-y

**Published:** 2023-03-15

**Authors:** Frederikke Lihme, Saima Basit, Lucca Katrine Sciera, Anne-Marie Nyboe Andersen, Henning Bundgaard, Jan Wohlfahrt, Heather A. Boyd

**Affiliations:** 1 Department of Epidemiology Research, Statens Serum Institute, Copenhagen, Denmark; 2 Section of Epidemiology Department of Public Health, The Faculty of Health Sciences, University of Copenhagen, Copenhagen, Denmark; 3 Department of Cardiology, University Hospital of Copenhagen (Rigshospitalet), Copenhagen, Denmark; 4 Statens Serum Institut, Building 206, Artillerivej 5, 2300 Copenhagen S, Denmark

**Keywords:** Cardiovascular disease, Epidemiology, Ischemic heart disease, Ischemic stroke, Myocardial infarction, Preeclampsia

## Abstract

Preeclampsia and cardiovascular disease (CVD) might share heritable underlying mechanisms. We investigated whether preeclampsia in daughters is associated with CVD in parents. In a register-based cohort study, we used Cox regression to compare rates of CVD (ischemic heart disease, ischemic stroke, myocardial infarction) in parents with ≥ 1 daughters who had preeclampsia and parents whose daughters did not have preeclampsia in Denmark, 1978–2018. Our cohort included 1,299,310 parents, of whom 87,251 had ≥ 1 daughters with preeclampsia and 272,936 developed CVD during 20,252,351 years of follow-up (incidence rate 135/10,000 person-years). Parents with one daughter who had preeclampsia were 1.19 times as likely as parents of daughters without preeclampsia to develop CVD at age < 55 years (hazard ratio [HR] 1.19, 95% confidence interval [CI] 1.13–1.25). Having ≥ 2 daughters who had preeclampsia yielded an HR of 1.88 (95% CI 1.39–2.53). The corresponding HRs for CVD at ≥ 55 years of age were 1.13 (95% CI 1.12–1.15) and 1.27 (95% CI 1.16–1.38). Patterns of association were similar for all CVD subtypes. Effect magnitudes did not differ for mothers and fathers (p = 0.52). Analyses by timing of preeclampsia onset in daughters suggested a tendency toward stronger associations with earlier preeclampsia onset, particularly in parents < 55 years. Preeclampsia in daughters was associated with increased risks of CVD in parents. Increasing strength of association with increasing number of affected daughters, equally strong associations for mothers and fathers, and stronger associations for CVD occurring before age 55 years suggest that preeclampsia and CVD share common heritable mechanisms.

**Supplementary Information:**

The online version contains supplementary material available at 10.1007/s10654-023-00972-y.

Women with a history of preeclampsia have greatly increased risks of cardiovascular disease (CVD) compared with women with no such history [1–7]. The links between preeclampsia and CVD are incompletely understood; the increased CVD risk observed post-preeclampsia could be due to behavioral risk factors or heritable predispositions common to both diseases, sequelae directly attributable to preeclampsia, or exacerbation of an existing predisposition to CVD by the additional cardiovascular system stress of preeclampsia [6].

Consistent with the suggestion that preeclampsia and CVD have overlapping features and vulnerabilities [6], we hypothesized that preeclampsia and CVD share common heritable mechanisms. Genetic predisposition to CVD before pregnancy might increase the risk of endothelial dysfunction, poor placentation, and organ dysfunction during pregnancy, contributing to preeclampsia risk [3, 8, 9]. Pregnancy could therefore represent a unique opportunity to identify women susceptible to vascular dysfunction and assess CVD risk in both the woman herself and her family. Conversely, CVD in parents might help to inform preeclampsia risk assessment in daughters.

We conducted a register-based cohort study to investigate whether preeclampsia and CVD co-aggregate in families. By examining the association between preeclampsia in daughters and CVD in parents, and considering the impact of multiple affected daughters, timing of preeclampsia onset, and timing of CVD in parents, we explored whether the diseases might share underlying risk factors and if so, whether common determinants of disease are most likely to be heritable, behavioral or both.

## Methods

### Study cohort

**Fig. 1:**
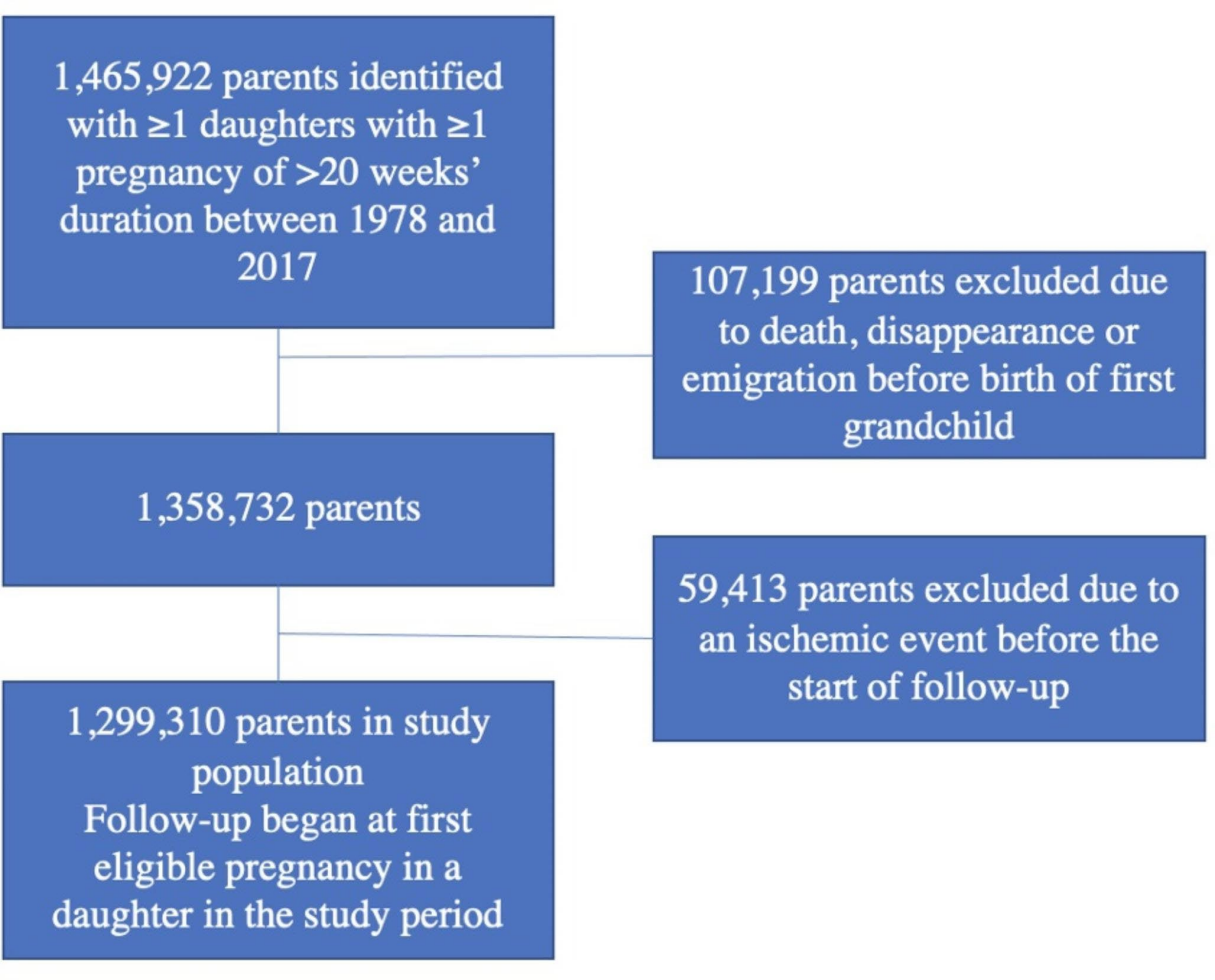
Study cohort assembly and exclusions

Using the Danish Civil Registration System, the National Patient Register and the Medical Birth Register (see eMethods, Data Sources, in the Supplement) [10–12], we identified all women in Denmark ≥ 15 years of age with one or more pregnancy of > 20 weeks’ duration between 1978 and 2017. We then identified each woman’s parents using the Danish Family Relations Database (see eMethods, Data Sources, in the Supplement); the parents constituted the study cohort. Parents who experienced one of the study outcomes before the first eligible pregnancy in a daughter in the study period were excluded (Fig. 1). The study was approved by Statens Serum Institut’s Compliance Department and registered with the Danish Data Protection Agency; under Danish law, neither informed consent nor ethics committee approval is required for strictly register-based studies.

### Number of daughters with preeclampsia (exposure)

The exposure of interest was the number of daughters with preeclampsia complicating one or more pregnancies. A daughter was considered to have preeclampsia in a given pregnancy if she was registered in the National Patient Register with preeclampsia, eclampsia, or the hemolysis, elevated liver enzymes and low platelets (HELLP) syndrome (see eMethods, Definitions, in the Supplement). We further classified preeclampsia according to gestational age at delivery (a proxy for timing of onset) into early preterm (delivery < 34 completed weeks’ gestation), late preterm (delivery between 34 and 36 completed weeks’ gestation) and term (delivery ≥ 37 completed weeks’ gestation) preeclampsia; hereafter, when we refer to timing of preeclampsia onset, we refer to this proxy classification. When we examined the importance of timing of preeclampsia onset in daughters, we used a variable combining the number of daughters with preeclampsia and the timing of preeclampsia onset in each daughter (see eMethods, Definitions, in the Supplement).

We considered number of daughters with preeclampsia as a time-dependent variable. A parent could contribute person-time to several exposure groups, changing exposure status from unexposed to exposed the first time a daughter had preeclampsia and changing exposure category with each additional daughter who experienced a pregnancy with preeclampsia. Once exposed, a parent could not become unexposed again.

### Cardiovascular disease in parents (outcome)

A parent was considered to have had CVD on the date of his/her first registration in the National Patient Register or Causes of Death Register with myocardial infarction, cerebrovascular infarction (ischemic stroke) or ischemic heart disease (not including myocardial infarction) (see eMethods, Definitions, in the Supplement). Parents contributed to the analyses with their earliest registered CVD, if any. If at first registration of CVD more than one type of event was registered and one of the events was a myocardial infarction, the parent was classified as having had a myocardial infarction. If no myocardial infarction was registered at first registration of CVD but both an ischemic stroke and ischemic heart disease were registered, one of the two events was selected at random. We further classified CVD as early-onset (< 55 years of age) and later-onset (≥ 55 years of age), because our evaluation of the proportional hazards assumption suggested that it might not be met for parental age. Although the American College of Cardiology defines premature heart disease as heart disease in women < 65 years of age and men < 55 years of age [13], we chose to use the 55-year cutoff for both sexes because residual plots (see Statistical analysis) suggested that 55 years would be a good cutoff for both groups and to simplify the comparison of estimates for fathers and mothers.

### Covariates

We considered parental birth year and sex (i.e., whether the parent was a mother or a father), number of daughters contributing pregnancies, parental diabetes (see eMethods, Definitions, in the Supplement) and total number of children to be potential confounders; parental sex and number of daughters contributing pregnancies were also evaluated as potential effect modifiers. We assumed that total number of children was a time-independent variable (i.e., that parents had had all their children by the start of follow-up) but treated number of daughters contributing with pregnancies as a time-dependent variable, because this number could increase during the follow-up period. We included parental birth year to help account for possible time trends in preeclampsia and CVD diagnoses. We did not adjust for hypertension in parents or daughters, as doing so would block the very link between preeclampsia in daughters and CVD in parents that we aimed to study, namely any association attributable to a heritable mechanism common to preeclampsia and CVD. (See the simplified acyclic directed graph and associated explanation in the eMethods in the Supplement for further clarification.)

### Statistical analysis

Parents were followed from the date of delivery in the first eligible pregnancy in a daughter to the first of the following events (competing risks analysis [14], see eMethods in the Supplement): (1) myocardial infarction; (2) ischemic stroke; (3) ischemic heart disease; (4) death due to non-cardiovascular causes; (5) emigration; (6) registration as “missing” in the Civil Registration System; or (7) 31 August 2018 (end of follow-up). Follow-up ended when a parent experienced one of the above events, regardless of whether they experienced an event of a different type later on. We used a cause-specific hazards model (Cox regression) with parental age as the underlying time scale to estimate hazard ratios comparing rates of CVD in parents with daughters with a history of preeclampsia and parents whose daughters had been pregnant but had no history of preeclampsia. The baseline hazards were stratified by parental birth year (five-year intervals), parental sex, number of daughters with pregnancies in the study period, and total number of children. We used Wald chi-squared tests across all exposure levels to determine whether hazard ratio magnitudes for the individual CVD types differed from one another. In a sensitivity analysis, we further adjusted the estimates for parental diabetes as a time-dependent variable.

Potential violations of the proportional hazards assumption were checked by plotting cumulative Martingale residuals [15]. We found that the assumption might not be met for parental age and that age 55 years was a good cutoff to use for stratification. Therefore, with the exception of results stratified by timing of preeclampsia onset (where power was an issue), we present results stratified by parental age (< 55 years and ≥ 55 years).

All analyses were performed using SAS version 9.4 (SAS Institute Inc., Cary, NC).

## Results

The cohort consisted of 1,299,310 persons (690,194 mothers and 609,116 fathers) who had one or more daughters with eligible pregnancies between 1978 and 2017 and who had not been registered with CVD before the start of follow-up (Fig. 1). Table 1 presents basic characteristics of the cohort at the start of follow-up. More mothers than fathers were < 50 years of age at the start of follow-up, whereas more fathers were ≥ 60 years of age. Almost half of all parents (46%) had two children; 13% had only one child, while 14% had four or more. The majority of parents (56%) had one daughter, 34% had two daughters, and only 10% had three or more daughters. (However, not all daughters became pregnant during the study period and therefore not all contributed to the analysis). During follow-up, 87,251 parents had one or more daughters with preeclampsia.

**Table 1:** Characteristics of parents of daughters with one or more pregnancies of at least 20 weeks’ duration in Denmark between 1978 and 2017

Characteristic	Number (%)		
	Mothers (N = 690,194)	Fathers (N = 609,116)	Total (N = 1,299,310)
Age at the start of follow-up (years)			
< 40	19,000 (2.8)	5,423 (0.9)	24,423 (1.9)
40–44	79,965 (11.6)	39,013 (6.4)	118,978 (9.2)
45–49	152,927 (22.2)	103,545 (17.0)	25,647 (19.7)
50–54	183,061 (26.5)	156,568 (25.7)	339,629 (26.1)
55–59	140,808 (20.4)	148,454 (24.7)	289,262 (22.3)
60–64	75,103 (10.9)	92,549 (15.2)	167,652 (12.9)
65–69	29,016 (4.2)	42,585 (7.0)	71,601 (5.5)
≥ 70	10,314 (1.5)	20,979 (3.4)	31,293 (2.4)
Number of daughters at the start of follow-up†			
1	391,928 (56.8)	336,766 (55.3)	728,694 (56.1)
2	230,923 (33.5)	206,351 (33.9)	437,274 (33.7)
3	55,245 (8.0)	53,200 (8.7)	108,445 (8.4)
≥ 4	12,098 (1.8)	12,799 (2.1)	24,897 (1.9)
Total number of children at the start of follow-up			
1	92,279 (13.8)	79,183 (13.0)	171,462 (13.2)
2	321,041 (46.5)	273,233 (44.9)	594,274 (45.7)
3	187,670 (27.2)	167,445 (27.5)	355,115 (27.3)
≥ 4	89,204 (12.9)	89,255 (14.7)	178,459 (13.7)
Diabetes at the start of follow-up			
Yes	9,877 (1.4)	12,920 (2,1)	22,797 (1.8)
No	680,317 (98.6)	596,196 (97.9)	1,276,513 (98.2)

† Including daughters not contributing with pregnancies

We followed the cohort for 20,252,351 person-years, during which 4,823 parents were lost to follow-up (128 were lost to the Civil Registration System and 4,695 emigrated). Median follow-up time was 16.0 years (interquartile range: 8.5–24.3 years) for mothers and 13.1 years (interquartile range: 6.7–20.7 years) for fathers. Overall, 272,936 parents were registered with CVD during follow-up (incidence rate 135/10,000 person-years). Among mothers with CVD (N = 113,645), 28,079 (24%) had a myocardial infarction as their first event (incidence rate 24.3/10,000 person-years), 28,499 (25%) had an ischemic stroke (incidence rate 24.6/10,000 person-years), and 57,067 (50%) had ischemic heart disease (incidence rate 49.3/10,000 person-years). The corresponding numbers for fathers (N = 159,291) were 54,879 (34%), 33,986 (21%), and 70,426 (44%) (incidence rates 63.3, 39.2 and 81.2 per 10,000 person-years), respectively.

**Fig. 2:**
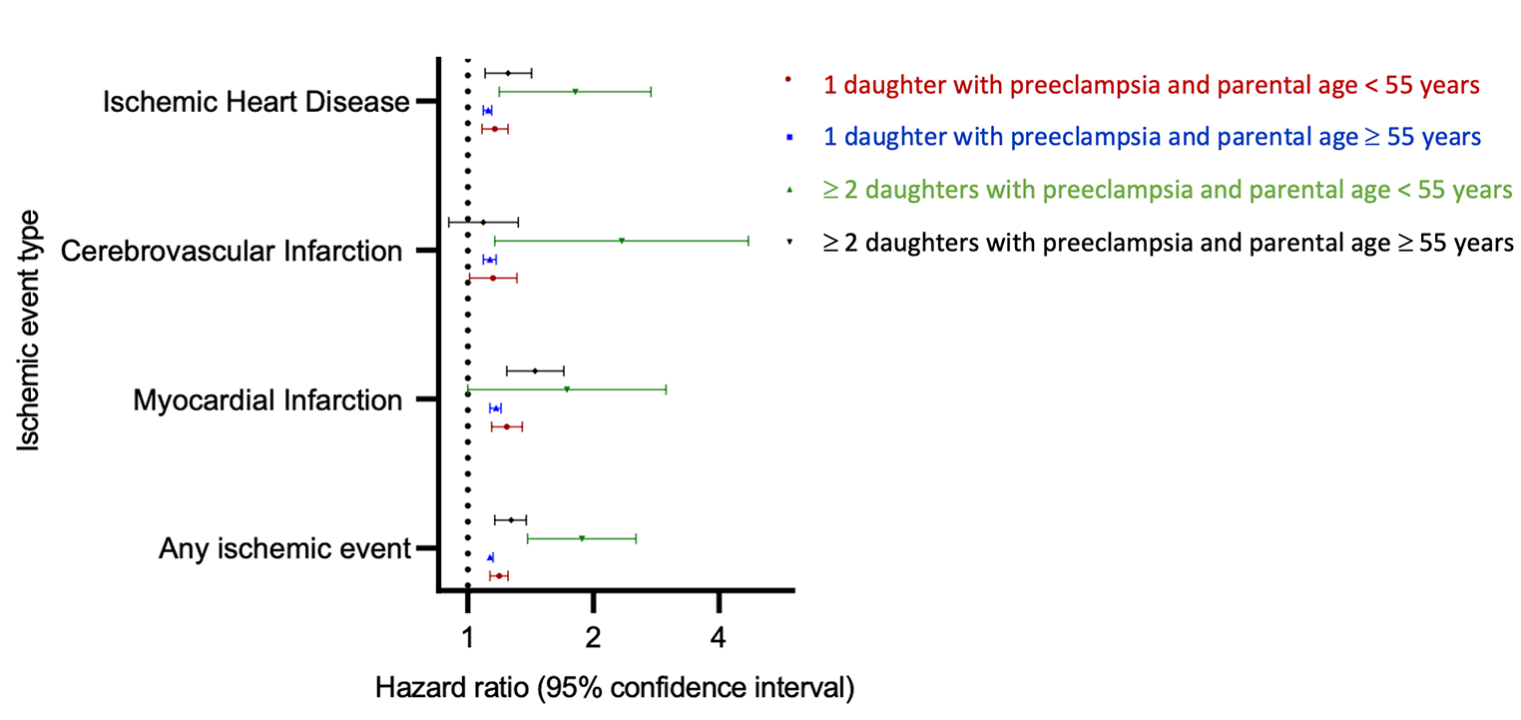
Hazard ratios for cardiovascular disease in parents by parental age, number of daughters with preeclampsia and type of cardiovascular disease. Hazard ratios for cardiovascular disease in parents at < 55 years of age with 1 affected daughter (red) and ≥2 affected daughters (green) and at ≥55 years of age with 1 affected daughter (blue) and ≥2 affected daughters (black), compared with parents with daughters with no history of preeclampsia (reference group). Hazard ratios were estimated in a cohort of parents with daughters having ≥1 pregnancies of at least 20 weeks’ duration in Denmark in the period of 1978–2017. All hazard ratios were estimated with the baseline hazards stratified by parental birth year (five-year intervals), parental sex, number of daughters with pregnancies in the study period, and total number of children. Age was the underlying time scale in the Cox model.

Having one daughter with a history of preeclampsia was associated with a 19% increase in the rate of any parental CVD at < 55 years of age, compared with having one daughter whose pregnancy history did not include preeclampsia (hazard ratio [HR] 1.19, 95% confidence interval [CI] 1.13–1.25) (Fig. 2, eTable 1). Having two or more daughters with a history of preeclampsia was more strongly associated with parental CVD in this age group (HR 1.88, 95% CI 1.39–2.53) (Fig. 2, eTable 1). The corresponding hazard ratios for any CVD in parents ≥ 55 years of age were 1.13 (95% CI 1.12–1.15) and 1.27 (95% CI 1.16–1.38), respectively. The number of daughters contributing to the analyses (i.e., the number of daughters a parent had who were pregnant during the study period) did not modify the strength of the associations between number of daughters with a history of preeclampsia and parental CVD (eTable 2, P for interaction = 0.12).

When we examined CVD types separately, the pattern of increasing strength of association with increasing number of daughters with preeclampsia appeared most pronounced for events occurring at < 55 years of age (Fig. 2, eTable 1). Hazard ratios for myocardial infarction at < 55 years increased from 1.24 (95% CI 1.14–1.35) for parents with one affected daughter to 1.73 (95% CI 1.00-2.99) for parents with two or more affected daughters, compared with parents whose daughters had no history of preeclampsia. The corresponding estimates for myocardial infarctions at ≥ 55 years of age were 1.17 (95% CI 1.13–1.20) and 1.45 (95% CI 1.24–1.70), respectively. A similar pattern was observed for ischemic stroke and ischemic heart disease (Fig. 2, eTable 1), although hazard ratios for ischemic stroke in parents > 55 years of age did not differ by number of affected daughters. Testing for differences in pattern across the three CVD types showed no differences (P = 0.80) by event type; however, estimates for the associations with preeclampsia in two or more daughters were based on relatively small numbers of parental events (Fig. 2, eTable 1).

**Fig. 3:**
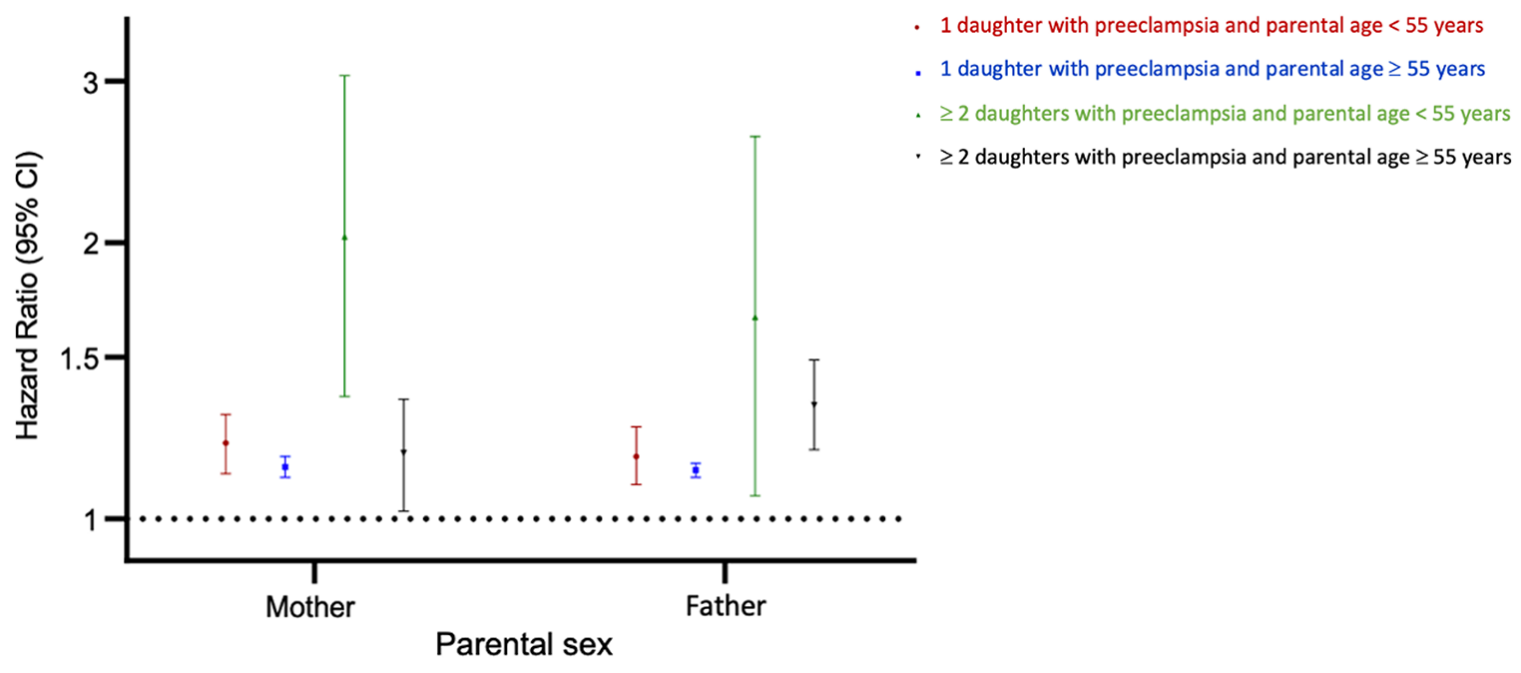
Hazard ratios for any cardiovascular disease in parents by parental sex, age and number of daughters. Hazard ratios for cardiovascular disease in parents at < 55 years of age with 1 affected daughter (red) and ≥2 affected daughters (green) and at ≥55 years of age with 1 affected daughter (blue) and ≥2 affected daughters (black), compared with parents with daughters with no history of preeclampsia (reference group). Hazard ratios were estimated in a cohort of parents with daughters having ≥1 pregnancies of at least 20 weeks’ duration in Denmark in the period of 1978–2017. All hazard ratios were estimated with the baseline hazards stratified by parental birth year (five-year intervals), number of daughters with pregnancies in the study period, and total number of children. Age was the underlying time scale in the Cox model.

Separate analyses by parental sex showed that associations for mothers and fathers did not differ from one another for any outcome (Fig. 3, eTables 3 A and B, all p-values ≥0.05). However, in mothers, the HRs for any CVD at < 55 years and at ≥ 55 years differed from one another (P = 0.02), whereas in fathers the estimates for the two age groups were similar (P = 0.44).

When we examined gestational age at preeclampsia onset in daughters, the results suggested that preterm preeclampsia, particularly in multiple daughters, might be most strongly associated with CVD in parents (Table 2). However, we often lacked power to detect differences across exposure groups and strata; in particular, there were few families with two or more daughters with early-onset preeclampsia and CVD among parents. Consequently, confidence intervals around most hazard ratios were very wide.

**Table 2:** Hazard ratios for combined cardiovascular disease in parents of daughters with one or more pregnancies of at least 20 weeks’ duration in Denmark between 1978 and 2017, by parental age, number of daughters with a history of preeclampsia and timing of preeclampsia onset in these daughters

Timing of preeclampsia onset†	Number of daughters with preeclampsia	Combined cardiovascular disease‡							
		Parental age < 55 years				Parental age ≥ 55 years			
		No. of events	No. of person-years (x10^3^)	HR§	95% CI	No. of events	No. of person-years (x10^3^)	HR§	95% CI
No preeclampsia	0	21,216	3,741.0	1	Ref	214,524	14,123.0	1	Ref
Early preterm (< 34 weeks)	1	131	14.5	1.46	1.23–1.74	1,166	68.6	1.16	1.10–1.23
	2	< 5	0.3	1.80	0.67–4.81	52	2.9	1.22	0.93–1.61
Late preterm (34–36 weeks)	1	175	23.0	1.28	1.10–1.48	1,812	107	1.16	1.10–1.21
	2	1,000	0.6	2.73	1.47–5.07	84	4.1	1.36	1.10–1.69
Term	1	1,174	175	1.15	1.09–1.22	12,861	748	1.13	1.11–1.15
	2	26	2.3	1.64	1.12–2.42	288	15.5	1.22	1.09–1.37
Any preeclampsia	≥ 3	< 5	0.05	2.70	0.38–19.3	17	0.7	1.40	0.87–2.26

CI, confidence interval. HR, hazard ratio

† The exposure variable is a single categorical variable combining the timing of preeclampsia and the number of affected daughters. Because the exposure variable was time-dependent, parents could contribute person-time to more than one exposure category. Because we did not have enough power to consider every possible combination separately, we combined some categories. Two daughters with preeclampsia: If at least one daughter had early preterm preeclampsia, the parent was classified as exposed to early preterm preeclampsia. If no daughter had early preterm preeclampsia and at least one daughter had late preterm preeclampsia, the parent was classified as exposed to late preterm preeclampsia. Three or more affected daughters: all preeclampsia combined, regardless of timing of onset

‡ Myocardial infarction, ICD-8 code 410 or ICD-10 codes I21-I23; ischemic stroke, ICD-8 codes 433.09, 433.99, 436.01, 436.09, 436.90 or 436.99 or ICD-10 code I63; ischemic heart disease, ICD-8 codes 411–414 or ICD-10 codes I20, I24 or I25

§ All hazard ratios were estimated with the baseline hazards stratified by parental birth year (five-year intervals), parental sex, number of daughters with pregnancies in the study period, and total number of children

Additional adjustment for parental diabetes affected hazard ratio magnitudes very little (eTable 4). The strength of the observed associations did not depend on daughters’ parity (P for interaction = 0.59, eTable 5). In a sensitivity analysis restricted to the first daughter contributing a pregnancy in the study period, adjustment for the daughter’s age at the start of follow-up changed the results very little (eTable 6). Finally, when we repeated the analyses using generalized estimating equations to account for potential correlation of CVD outcomes within families (to avoid the possibility that the observed associations were driven by the co-aggregation of preeclampsia and CVD within a few large families), HRs and CIs were identical (out to the fourth decimal place) to those obtained in our main analyses (data not shown).

## Discussion

In a cohort of almost 1.3 million parents, preeclampsia in daughters was associated with an increased parental risk of CVD, especially for CVD occurring before age 55 years. The associations were equally strong for mothers and fathers. The strength of the associations increased markedly with an increasing number of affected daughters for all events except ischemic stroke at ≥ 55 years of age, suggesting a possible dose-response relationship. However, there were too few ischemic strokes at ≥ 55 years of age among persons whose daughters had had preeclampsia to allow us to conclude that the pattern of association differed for different CVD types. The association with preeclampsia in two or more daughters appeared to be particularly strong for parental CVD occurring before 55 years of age. Our results also suggested that preeclampsia in daughters with onset at earlier gestational ages might be most strongly associated with parental CVD.

Many studies have linked preeclampsia with increased risk of later CVD [2, 4, 5, 16, 17]. Less is known about potential familial predisposition to both conditions [18]. Of previous studies investigating family history of CVD as a risk factor for preeclampsia, two found no association [19, 20], whereas five reported odds ratios between 1.58 and 3.65 [21–25]. The studies ascertained family history of CVD (often in any relative and at any age) via interview either during pregnancy or retrospectively, once the outcome of pregnancy was known, which might have introduced significant recall bias. No study has previously investigated offspring preeclampsia as a risk factor for CVD in parents.

Previous studies were not designed to determine whether preeclampsia and CVD are linked by shared predispositions or preeclampsia causes de novo cardiovascular damage that results in CVD [2, 9, 18, 26, 27]. Our study results favor the former, as preeclampsia in a daughter cannot cause CVD in a parent. The finding of equal strength of association for mothers and fathers supports the hypothesis that preeclampsia is linked with CVD through common heritable factors. Stronger associations for mothers than for fathers could have been explained by the known, strong familial aggregation of preeclampsia in the female line (the paternal contribution is decidedly smaller [28]) coupled with mothers’ increased risk of CVD after their own preeclampsia. Our findings that parental risk of CVD increased with the number of daughters with preeclampsia, associations with multiple affected daughters were stronger for early CVD in parents than for later events, and associations might be stronger for early-onset preeclampsia in daughters, all further support a role for common heritable factors in explaining the associations. CVD later in life and late-onset preeclampsia share common risk factors with a behavioral component, including overweight, the metabolic syndrome, and diabetes, which might suggest that such factors could drive the observed associations if the conditions also clustered strongly in the same families. However, early CVD and preeclampsia with onset < 37 weeks are both much less strongly associated with the above-mentioned conditions and other behavioral risk factors.

While our findings do not exclude contributions from shared behavioral factors, they indicate a role for heritable genetic mechanisms common to preeclampsia and CVD. Genome-wide association studies of early-onset preeclampsia have identified genetic variants shared by the two conditions [8], suggesting that joint susceptibility to preeclampsia and CVD might be mediated by inherited predisposition to hypertension, inflammation, endothelial dysfunction or other abnormalities resulting in vascular damage, rather than predisposition to more behaviorally contingent factors such as overweight and diabetes [29–31].

The inclusion of the entire Danish population in the cohort minimized the risk of selection bias, and we addressed previous studies’ potential problems with recall bias by using prospectively collected data on both preeclampsia and CVD and restricting to outcomes in parents. The large study population and long follow-up period provided us with excellent power to examine outcomes temporally far removed from the exposure, examine associations with individual ischemic outcomes, and categorize exposure and outcome by timing of onset, which has not previously been done.

Registration of myocardial infarctions and ischemic strokes in the National Patient Register is virtually complete and validation of registered diagnoses against medical records found positive predictive values exceeding 92% and 97%, respectively [11, 32]. Registration of ischemic heart disease, which can initially be diagnosed by general practitioners (who do not report to the National Patient Register), is probably less complete or at least delayed, but registered diagnoses have not been validated. Registration of preeclampsia is incomplete, with a sensitivity of 69.3% overall [33]. However, the specificity exceeds 99% for all preeclampsia subtypes, indicating that registered diagnoses overwhelmingly reflect true instances of preeclampsia. Due to the excellent specificity of registered preeclampsia and CVD diagnoses, the effect of any bias due to misclassification on our results was likely negligible.

We could not adjust for certain comorbidities that can aggregate in families and are associated with both preeclampsia and CVD, including the metabolic syndrome, overweight, and maternal preeclampsia. Registration of metabolic syndrome diagnoses only began recently; BMI is only registered in connection with pregnancy and then only since 2003. Registration of pregnancy complications in the National Patient Register began in 1978; consequently, only a minority of mothers in the study cohort had their own pregnancy experience registered and a sensitivity analysis restricted to this small group of mothers would not have had meaningful statistical power. The finding of a similar pattern of association in fathers, however, indicates that clustering of preeclampsia in the maternal line cannot explain the familial co-aggregation of preeclampsia and CVD.

We also lacked the information to allow us to adjust for potential confounding by shared familial socioeconomic and lifestyle factors. How and to what extent such factors might have been shared trans-generationally in our study population is difficult to assess, making it challenging to determine how any residual confounding by these factors might have affected the observed results. Confounding by shared socioeconomic and lifestyle factors would be expected to be independent of parental age, whereas we observed stronger associations in parents at younger ages (< 55 years) than in older parents. This suggests that the associations in younger parents are more likely to be chiefly related to genetic factors than to be the product of residual confounding by socioeconomic and lifestyle factors. However, the associations observed in older parents may well be partially explained by such factors. While the results of this study have no immediate implications for clinical practice, they suggest potential modifications to risk assessment practices for both preeclampsia and CVD. Future research should determine whether knowledge of preeclampsia history in daughters could help identify persons at increased risk of CVD, potentially by testing the effect of adding this information to existing algorithms [34, 35] for assessing CVD risk. Conversely, new research could also investigate whether knowledge of early-onset CVD in a woman’s parents might improve prediction of preeclampsia risk, which is especially difficult in nulliparous women.

Having one or more daughters with a history of preeclampsia was associated with increases in the risk of CVD in parents. Associations were particularly pronounced for parental CVD occurring before 55 years of age. Our findings suggest that preeclampsia and CVD share common heritable mechanisms.

## Electronic supplementary material

Below is the link to the electronic supplementary material.

Supplementary Material 1

## Data Availability

The data on which this study are based do not belong to the authors but to the Danish Health Data Authority, and the authors are therefore not permitted to make the data publicly available. However, upon receipt of approval from the Danish Data Protection Agency, data from the Danish national health registers are available upon application through an online proposal system that can be found on the Danish Health Data Authority’s website (https://sundhedsdatastyrelsen.dk/da/registre-og-services).
