# Supplementary material for: Association between preeclampsia in daughters and risk of cardiovascular disease in parents
Source: Eur J Epidemiol. 2023 Mar 15;38(3):335–43. doi: 10.1007/s10654-023-00972-y (PMC10033554; doi:10.1007/s10654-023-00972-y)
Supplement: Supplementary file 1 — Supplementary Material 1 [file 10654_2023_972_MOESM1_ESM.docx]

**SUPPLEMENTARY CONTENT**

**Association between preeclampsia in daughters and risk of cardiovascular disease in parents**

Frederikke Lihme, MD, Saima Basit, MSc, Lucca K. Sciera, MPH, Anne-Marie Nyboe Andersen, MD, PhD, Henning Bundgaard, MD, DMSc, Jan Wohlfahrt, DMSc, Heather A. Boyd, PhD

**INDEX**

1. **eMethods**

Data Sources

Definitions

Competing Risks and Censoring

Simplified Directed Acyclic Graph Supporting the Decision not to Consider Hypertension as a Confounder

1. **eTables**

eTable 1. Hazard ratios for myocardial infarction, ischemic stroke, and ischemic heart disease by parental age and number of daughters with preeclampsia, in parents of daughters with one or more pregnancies of at least 20 weeks’ duration in Denmark, 1978-2017

eTable 2. Hazard ratios for any cardiovascular disease by number of daughters with a history of preeclampsia and number of daughters contributing pregnancies to the analysis, in parents of daughters with one or more pregnancies of at least 20 weeks’ duration in Denmark, 1978-2017

eTable 3A. Hazard ratios for myocardial infarction, stroke and ischemic heart disease at <55 years of age by number of daughters with a history of preeclampsia, separately for mothers and fathers of daughters with one or more pregnancies of at least 20 weeks’ duration in Denmark, 1978-2017

eTable 3B. Hazard ratios for myocardial infarction, stroke and ischemic heart disease at ≥55 years of age by number of daughters with a history of preeclampsia, separately for mothers and fathers of daughters with one or more pregnancies of at least 20 weeks’ duration in Denmark, 1978-2017

eTable 4. Hazard ratios for myocardial infarction, ischemic stroke, and ischemic heart disease by parental age and number of daughters with preeclampsia, additionally adjusted for parental diabetes, in parents of daughters with one or more pregnancies of at least 20 weeks’ duration in Denmark, 1978-2017.

eTable 5. Hazard ratios for any cardiovascular disease by attained age and number of daughters with preeclampsia in parents of daughters with one or more pregnancies of at least 20 weeks’ duration in Denmark, 1978-2017, stratified by daughters’ parity

eTable 6. Hazard ratios for any cardiovascular disease by attained age in parents of daughters with one or more pregnancies of at least 20 weeks’ duration in Denmark, 1978-2017 when the analysis was restricted to the first pregnancy in the first daughter contributing a pregnancy during the study period, with additional adjustment for the daughter’s age at delivery

1. **eReferences**
2. **eMETHODS**

**DATA SOURCES**

The study cohort was constructed using data from the **Danish Civil Registration System**. The Danish Civil Registration System is a national population register established in 1968 that contains demographic and kinship information on all Danish residents. All persons registered in the system have a unique identification number which enables accurate linkage of data from all national registers^1,2^. Contacts with the national healthcare system are registered using the personal identification number, which makes it possible to conduct studies based on Danish registers with minimal loss to follow-up.

Based on kinship information from the Civil Registration System, Statens Serum Institut created the **Danish Family Relations Database,** a database of kinship ties in the Danish population.

The **National Patient Register** contains information on all in- and outpatient visits to public hospitals in Denmark since 1977. It registers events using International Classification of Disease (ICD) codes, version 8 (ICD-8) from 1977-1993 and version 10 (ICD-10) codes thereafter^3^.

The **Causes of Death Register** contains information on all deaths among Danish residents dying in Denmark. The register dates back to 1970 and registers date of death, underlying cause of death, and contributing causes of death, classified using ICD codes^4,5^.

The **Medical Birth Register** contains information on all births in Denmark from 1973, including information on the pregnancy, the delivery, the outcome of the birth, and any pregnancy or delivery complications^6^.

**DEFINITIONS**

Preeclampsia

Preeclampsia is currently defined as incident hypertension with onset after 20 weeks of gestation, accompanied by proteinuria and/or other signs of organ dysfunction^7,8^. However, in the study period, an older definition was used. Preeclampsia diagnoses registered in the National Patient Register were coded according to Danish Society of Obstetrics and Gynecology (DSOG) guidelines^9^, using the following codes: ICD-8: 637.04, 637.03, 637.09, 637.99, 762.19, 762.29, 762.39; ICD-10: O14.0, O.14.1, O14.2, O14.9. Mild/moderate preeclampsia was defined as hypertension accompanied by proteinuria; women who also had severe hypertension and/or severe proteinuria and/or signs of organ failure (including the HELLP syndrome) were registered with severe preeclampsia. Women who also had generalized seizures were registered eclampsia (ICD-10: O15.0-15.9), which was grouped with preeclampsia in our study due to the small number of women suffering from this condition in Denmark.

To ensure that registered diagnoses reflected true cases of preeclampsia, women whose only diagnoses were registered more than 1 month before delivery or more than 7 days postpartum were not considered to have had preeclampsia. The definition was designed to catch probable false positive cases of preeclampsia arising in the instance of women evaluated for preeclampsia mid-pregnancy who received a conditional diagnosis that was later ruled out and who then proceed with an uncomplicated pregnancy ending in delivery at term, with the preeclampsia diagnosis still on the books. A woman with preeclampsia will visit the hospital regularly (or be admitted), with the diagnosis re-registered at every visit. Clinical guidelines also require re-registration of a preeclampsia diagnosis immediately postpartum. It is therefore very unlikely that a woman with true preeclampsia would not have the diagnoses registered in the month prior to delivery or within a week of delivery but would have diagnoses registered earlier in her pregnancy. Thus, while the restriction might create a slight decrease in the sensitivity of our definition of preeclampsia, the increase in positive predictive value outweighs it.

We defined early preterm preeclampsia as preeclampsia in a woman who delivered before 34 completed weeks’ gestation, late preterm preeclampsia as preeclampsia with delivery at 34-36 completed weeks’ gestation, and term preeclampsia as preeclampsia with delivery at or after 37 completed weeks’ gestation.

In analyses stratified by gestational age at onset of preeclampsia (Table 2), there were 10 possible exposure groups for parents with daughters with preeclampsia:

1.    1 daughter with early preterm preeclampsia

2.    1 daughter with late preterm preeclampsia

3. 1 daughter with term preeclampsia

4. 2 daughters with early preterm preeclampsia

5. 2 daughters with late preterm preeclampsia

6. 2 daughters with term preeclampsia

7.    1 daughter with early preterm preeclampsia and 1 daughter with late preterm preeclampsia

8.    1 daughter with early preterm preeclampsia and 1 daughter with term preeclampsia

9.    1 daughter with late preterm preeclampsia and 1 daughter with term preeclampsia

10.  ≥3 daughters with preeclampsia, any timing of onset

For the analysis presented in the paper (Table 2), we combined some categories to improve power, yielding the following exposure group for parents with daugthers with preeclampsia:

1. 1 daughter with early preterm preeclampsia
2. 1 daughter with late preterm preeclampsia
3. 1 daughter with term preeclampsia
4. 2 daughters with preeclampsia, at least one of whom had early preterm preeclampsia
5. 2 daughters with preeclampsia, neither with early preterm preeclampsia, at least one of whom had late preterm preeclampsia
6. 2 daughters with preeclampsia, both of whom had term preeclampsia
7. 3 or more daughters with preeclampsia, any type

Cardiovascular disease

We defined cardiovascular disease as registration in the National Patient Register or Causes of Death Register with the following ICD codes:

Myocardial infarction: ICD-8, 410; ICD-10, I21.0-I23.9

Stroke: ICD-8, 433.09, 433.99, 436.01, 436.09, 436.90 or 436.99; ICD-10, I63.0-63.9

Ischemic heart disease: ICD-8, 411-414; ICD-10, I20.0-20.9, I24.0-I25.9

Parental diabetes

We defined diabetes as registration in the National Patient Register with any of the following ICD codes: ICD-8, 250, 251; ICD-10, E10.0-E11.9

**COMPETING RISKS AND CENSORING**

We performed a competing risks analysis with censoring at the first of seven possible events. In Denmark, vital status and disease diagnoses are updated daily and all seven possible events are registered with exact dates (including date of emigration and date of last known contact for the few hundred truly lost to the Danish Civil Registration System). As a result, we do not have loss to follow up with need for interval censoring.

**SIMPLIFIED DIRECTED ACYCLIC GRAPH SUPPORTING THE DECISION NOT TO CONSIDER HYPERTENSION AS A CONFOUNDER**

Hypertension in parents

Cardiovascular disease

in parents

Heritable mechanism

Preeclampsia in daughters

Hypertension in

daughters

Preeclampsia is associated with later hypertension and cardiovascular disease (in the same woman). We – and others – hypothesize that the common factor linking these conditions likely involves increased susceptibility to vascular damage and/or inflammation and that this susceptibility is heritable. Preeclampsia is therefore a marker of increased familial risk of hypertension and cardiovascular disease. As is illustrated in the above simplified DAG, we hypothesized that the underlying heritable factor is independently associated with hypertension in parents, hypertension in daughters, and preeclampsia in daughters. In this situation, adjusting for hypertension in daughters or parents would block the very link between the underlying heritable factor, preeclampsia in daughters, and cardiovascular disease in parents that we aimed to study. As a result, we believe that in the context of our study, it would be a mistake to adjust for hypertension.

1. **eTABLES**

**eTable 1. Hazard ratios for myocardial infarction, ischemic stroke, and ischemic heart disease by attained age and number of daughters with preeclampsia in parents of daughters with one or more pregnancies of at least 20 weeks’ duration in Denmark, 1978-201****7**

| **Type of cardiovascular disease** | **No. daughters with PE** | **Age < 55 years** | | | | | **Age ≥55 years** | | | | |
| --- | --- | --- | --- | --- | --- | --- | --- | --- | --- | --- | --- |
|  |  | **No. events**† | **No. person-years (x10^3^)** | **CVD incidence per 10,000 person-years** | **HR**‡ | **95% CI** | **No. events**† | **No. person-years (x10^3^)** | **CVD incidence per 10,000 person-years** | **HR**‡ | **95% CI** |
| Myocardial infarction | 0 | 7,691 | 3,941.8 | 19.5 | 1 | Ref | 69,480 | 15,071.0 | 46.1 | 1 | Ref |
|  | 1 | 548 | 223.5 | 24.5 | 1.24 | 1.14-1.35 | 5,069 | 987.2 | 51.3 | 1.17 | 1.13-1.20 |
|  | ≥2 | 13 | 3.4 | 38.1 | 1.73 | 1.00-2.99 | 157 | 25.5 | 61.7 | 1.45 | 1.24-1.70 |
| Ischemic stroke | 0 | 3,542 | 3,941.8 | 8.9 | 1 | Ref | 54,577 | 15,071.0 | 36.2 | 1 | Ref |
|  | 1 | 236 | 223.5 | 10.6 | 1.15 | 1.01-1.31 | 4,019 | 987.2 | 40.7 | 1.13 | 1.09-1.17 |
|  | ≥2 | 8 | 3.4 | 23.5 | 2.34 | 1.16-4.70 | 103 | 25.5 | 40.5 | 1.09 | 0.90-1.32 |
| Ischemic heart disease | 0 | 11,113 | 3,941.8 | 28.2 | 1 | Ref | 107,487 | 15,071.0 | 71.3 | 1 | Ref |
|  | 1 | 767 | 223.5 | 34.3 | 1.16 | 1.08-1.25 | 7,867 | 987.2 | 79.7 | 1.12 | 1.09-1.14 |
|  | ≥2 | 22 | 3.4 | 64.6 | 1.81 | 1.19-2.75 | 237 | 25.5 | 93.1 | 1.25 | 1.10-1.42 |
| Any cardiovascular disease | 0 | 22,346 | 3,941.8 | 56.7 | 1 | Ref | 231,544 | 15,071.0 | 153.6 | 1 | Ref |
|  | 1 | 1,551 | 223.5 | 69.4 | 1.19 | 1.13-1.25 | 16,955 | 987.2 | 171.7 | 1.13 | 1.12-1.15 |
|  | ≥2 | 43 | 3.4 | 126.2 | 1.88 | 1.39-2.53 | 497 | 25.5 | 195.2 | 1.27 | 1.16-1.38 |

CI, confidence interval. CVD, cardiovascular disease. HR, hazard ratio. PE, preeclampsia.

† Myocardial infarction, ICD-8 code 410 or ICD-10 codes I21-I23; ischemic stroke, ICD-8 codes 433.09, 433.99, 436.01, 436.09, 436.90 or 436.99 or ICD-10 code I63; ischemic heart disease, ICD-8 codes 411-414 or ICD-10 codes I20, I24 or I25.

‡ All hazard ratios were estimated with the baseline hazards stratified by parental birth year (five-year intervals), parental sex, number of daughters with pregnancies in the study period, and total number of children.

Testing for differences in pattern across the three cardiovascular disease types using Wald Chi-square test showed no significant differences (P=0.80) by event type.

**eTable 2. Hazard ratios for any cardiovascular disease by number of daughters with a history of preeclampsia and number of daughters contributing pregnancies to the analysis, in parents of daughters with one or more pregnancies of at least 20 weeks’ duration in Denmark, 1978-2017**

| **Number of daughters contributing pregnancies** | **Number of daughters with preeclampsia** | **Number of events**† | **Number of person-years (x10^3^)** | **Hazard ratio**‡ | **95% confidence interval** |
| --- | --- | --- | --- | --- | --- |
| 1 | 0 | 190,934 | 14,694.3 | 1 | Ref |
|  | 1 | 10,973 | 746.0 | 1.15 | 1.13 -1.17 |
|  |  |  |  |  |  |
| 2 | 0 | 52,332 | 3,684.0 | 1 | Ref |
|  | 1 | 5,799 | 368.1 | 1.13 | 1.10- 1.16 |
|  | 2 | 343 | 19.0 | 1.34 | 1.21 -1.49 |
|  |  |  |  |  |  |
| ≥ 3 | 0 | 10,624 | 635 | 1 | Ref |
|  | 1 | 1,734 | 97.0 | 1.09 | 1.03 -1.14 |
|  | 2 | 178 | 9.1 | 1.21 | 1.04 -1.40 |
|  | 3 | 19 | 0.8 | 1.45 | 0.93-2.28 |

† Cardiovascular disease: myocardial infarction, ICD-8 code 410 or ICD-10 codes I21-I23; ischemic stroke, ICD-8 codes 433.09, 433.99, 436.01, 436.09, 436.90 or 436.99 or ICD-10 code I63; ischemic heart disease, ICD-8 codes 411-414 or ICD-10 codes I20, I24 or I25.

‡ All hazard ratios were estimated with the baseline hazards stratified by parental birth year (five-year intervals), parental sex, number of daughters with pregnancies in the study period, and total number of children.

Test of interaction between number of daughters contributing pregnancies and number of daughters with preeclampsia showed no significant interaction: P = 0.12.

**eTable 3A. Hazard ratios for myocardial infarction, stroke and ischemic heart disease at <55 years of age by number of daughters with a history of preeclampsia, separately for mothers and fathers of daughters with one or more pregnancies of at least 20 weeks’ duration in Denmark, 1978-2017**

| **No. of daughters with preeclampsia** | **No. of**  **person-years (x10^3^**) | **Myocardial infarction**† | | | **Ischemic stroke**† | | | **Ischemic heart disease** † | | | **Any cardiovascular disease**† | | |
| --- | --- | --- | --- | --- | --- | --- | --- | --- | --- | --- | --- | --- | --- |
|  |  | No. of events | HR‡ | 95% CI | No. of events | HR‡ | 95% CI | No. of events | HR‡ | 95% CI | No. of events | HR‡ | 95% CI |
| Mothers |  |  |  |  |  |  |  |  |  |  |  |  |  |
| 0 | 2,490.6 | * | 1 | Ref | 1,818 | 1 | Ref | 5,679 | 1 | Ref | 9,923 | 1 | Ref |
| 1 | 143.2 | 184 | 1.27 | 1.09-1.47 | 142 | 1.31 | 1.11-1.56 | 397 | 1.15 | 1.04-1.28 | 723 | 1.21 | 1.12-1.30 |
| ≥2 | 2.3 | * | 1.39 | 0.52-3.72 | 6 | 2.94 | 1.31-6.61 | 14 | 2.03 | 1.20-3.44 | 24 | 2.03 | 1.36-3.04 |
| Fathers |  |  |  |  |  |  |  |  |  |  |  |  |  |
| 0 | 1,451.2 | 5265 | 1 | Ref | * | 1 | Ref | 60,117 | 1 | Ref | 12,423 | 1 | Ref |
| 1 | 80.2 | 364 | 1.23 | 1.10-1.37 | 94 | 0.97 | 0.79-1.19 | 4,365 | 1.18 | 1.06 | 828 | 1.17 | 1.09-1.26 |
| ≥2 | 1.1 | 9 | 1.92 | 0.99-3.70 | * | 1.45 | 0.36-5.84 | 132 | 1.48 | 0.73-2.96 | 19 | 1.66 | 1.06-2.61 |

* To avoid potential identification of persons in categories with numbers < 5, some numbers will be hidden.

CI, confidence interval. HR, hazard ratio. PE, preeclampsia.

† Cardiovascular disease: myocardial infarction, ICD-8 code 410 or ICD-10 codes I21-I23; ischemic stroke, ICD-8 codes 433.09, 433.99, 436.01, 436.09, 436.90 or 436.99 or ICD-10 code I63; ischemic heart disease, excluding myocardial infarction, ICD-8 codes 411-414 or ICD-10 codes I20, I24 or I25.

‡ All hazard ratios were estimated with the baseline hazards stratified by parental birth year (five-year intervals), parental sex, number of daughters with pregnancies in the study period, and total number of children.

Wald Chi-squared test for difference of hazard ratios between sexes overall within this age strata showed no significant difference: P=0.60. Looking at events separately showed no differences between the sexes for any event (myocardial infarction: p=0.81, ischemic stroke: p=0.05, ischemic heart disease: p=0.78).

Wald Chi-squared test for difference of hazard ratios between age groups for any cardiovascular disease: mothers p=0.02, fathers p=0.44.

**eTable 3B. Hazard ratios for myocardial infarction, stroke and ischemic heart disease for parents at or above 55 years of age by number of daughters with a history of preeclampsia, separately for mothers and fathers of daughters with one or more pregnancies of at least 20 weeks’ duration in Denmark, 1978-2017**

| **No. of daughters with preeclampsia** | **No. of**  **person-years (x10^3^**) | **Myocardial infarction**† | | | **Ischemic stroke**† | | | **Ischemic heart disease** † | | | **Any cardiovascular disease**† | | |
| --- | --- | --- | --- | --- | --- | --- | --- | --- | --- | --- | --- | --- | --- |
|  |  | No. of events | HR‡ | 95% CI | No. of events | HR‡ | 95% CI | No. of events | HR‡ | 95% CI | No. of events | HR‡ | 95% CI |
| Mothers |  |  |  |  |  |  |  |  |  |  |  |  |  |
| 0 | 8,369.3 | 23,594 | 1 | Ref | 24,668 | 1 | Ref | 47,370 | 1 | Ref | 95,632 | 1 | Ref |
| 1 | 555.5 | 1820 | 1.21 | 1.15-1.27 | 1,821 | 1.12 | 1.07-1.18 | 3,502 | 1.11 | 1.07-1.15 | 7,143 | 1.14 | 1.11-1.17 |
| ≥2 | 14.7 | 51 | 1.32 | 1.00-1.74 | 44 | 1.01 | 0.75-1.36 | 105 | 1.20 | 0.99-1.45 | 200 | 1.18 | 1.02-1.35 |
| Fathers |  |  |  |  |  |  |  |  |  |  |  |  |  |
| 0 | 6,701.6 | 45,886 | 1 | Ref | * | 1 | Ref | 60,117 | 1 | Ref | 135,912 | 1 | Ref |
| 1 | 431.8 | 3,249 | 1.14 | 1.10-1.18 | 2,198 | 1.13 | 1.09-1.19 | 4,365 | 1.12 | 1.09-1.16 | 9,812 | 1.13 | 1.11-1.15 |
| ≥2 | 10.8 | 106 | 1 | Ref | * | 1 | Ref | 132 | 1 | Ref | * | 1 | Ref |

* To avoid potential identification of persons in categories with numbers < 5, some numbers will be hidden.

CI, confidence interval. HR, hazard ratio. PE, preeclampsia.

† Cardiovascular disease: myocardial infarction, ICD-8 code 410 or ICD-10 codes I21-I23; ischemic stroke, ICD-8 codes 433.09, 433.99, 436.01, 436.09, 436.90 or 436.99 or ICD-10 code I63; ischemic heart disease, excluding myocardial infarction, ICD-8 codes 411-414 or ICD-10 codes I20, I24 or I25.

‡ All hazard ratios were estimated with the baseline hazards stratified by parental birth year (five-year intervals), number of daughters with pregnancies in the study period, and total number of children.

Wald Chi-squared test for difference of hazard ratios between sexes overall within this age strata showed no significant difference: P=0.48. Looking at events separately showed no differences between the sexes for any event (myocardial infarction: p=0.09, ischemic stroke: p=0.71, ischemic heart disease: p=0.88).

Wald Chi-squared test for difference of hazard ratios between age groups for any cardiovascular disease: mothers p=0.02, fathers p=0.44.

**eTable 4. Hazard ratios for myocardial infarction, ischemic stroke, and ischemic heart disease by parental age and number of daughters with preeclampsia, additionally adjusted for parental diabetes, in parents of daughters with one or more pregnancies of at least 20 weeks’ duration in Denmark, 1978-2017.**

|  |  | **Age <55 years** | | | | | | | **Age ≥55 years** | | | | | |
| --- | --- | --- | --- | --- | --- | --- | --- | --- | --- | --- | --- | --- | --- | --- |
|  |  | |  |  | **Standard adjustments**‡ | | **Additionally adjusted for parental diabetes** | |  |  | **Standard adjustments**‡ | | **Additionally adjusted for parental diabetes** | |
| **Type of cardiovascular disease** | **No. daughters with PE** | | **No. events**† | **No. person-years (x10^3^)** | **HR** | **95% CI** | **HR*** | **95% CI** | **No. events**† | **No. person-years (x10^3^)** | **HR** | **95% CI** | **HR*** | **95% CI** |
| Myocardial infarction | 0 | | 7,691 | 3,941.8 | 1 | Ref | 1 | Ref | 69,480 | 15,071.0 | 1 | Ref | 1 | Ref |
|  | 1 | | 548 | 223.5 | 1.24 | 1.14-1.35 | 1.23 | 1.13-1.34 | 5,069 | 987.2 | 1.17 | 1.13-1.20 | 1.15 | 1.12-1.18 |
|  | ≥2 | | 13 | 3.4 | 1.73 | 1.00-2.99 | 1.71 | 0.99-2.96 | 157 | 25.5 | 1.45 | 1.24-1.70 | 1.41 | 1.20-1.65 |
| Ischemic stroke | 0 | | 3,542 | 3,941.8 | 1 | Ref | 1 | Ref | 54,577 | 15,071.0 | 1 | Ref | 1 | Ref |
|  | 1 | | 236 | 223.5 | 1.15 | 1.01-1.31 | 1.14 | 1.00-1.30 | 4,019 | 987.2 | 1.13 | 1.09-1.17 | 1.11 | 1.08-1.15 |
|  | ≥2 | | 8 | 3.4 | 2.34 | 1.16-4.70 | 2.29 | 1.14-4.62 | 103 | 25.5 | 1.09 | 0.90-1.32 | 1.06 | 0.87-1.29 |
| Ischemic heart disease | 0 | | 11,113 | 3,941.8 | 1 | Ref | 1 | Ref | 107,487 | 15,071.0 | 1 | Ref | 1 | Ref |
|  | 1 | | 767 | 223.5 | 1.16 | 1.08-1.25 | 1.15 | 1.07-1.24 | 7,867 | 987.2 | 1.12 | 1.09-1.14 | 1.10 | 1.07-1.12 |
|  | ≥2 | | 22 | 3.4 | 1.81 | 1.19-2.75 | 1.80 | 1.18-2.74 | 237 | 25.5 | 1.25 | 1.10-1.42 | 1.21 | 1.07-1.38 |
| Any cardiovascular disease | 0 | | 22,346 | 3,941.8 | 1 | Ref | 1 | Ref | 231,544 | 15,071.0 | 1 | Ref | 1 | Ref |
|  | 1 | | 1,551 | 223.5 | 1.19 | 1.13-1.25 | 1.17 | 1.12-1.24 | 16,955 | 987.2 | 1.13 | 1.12-1.15 | 1.12 | 1.10-1.13 |
|  | ≥2 | | 43 | 3.4 | 1.88 | 1.39-2.53 | 1.86 | 1.38-2.51 | 497 | 25.5 | 1.27 | 1.16-1.38 | 1.23 | 1.13-1.34 |

CI, confidence interval. HR, hazard ratio. PE, preeclampsia.

*Estimates additionally adjusted for parental diabetes (ICD-8: 250 and 251, ICD-10: E10 and E11).

† Any cardiovascular disease including: Myocardial infarction, ICD-8 code 410 or ICD-10 codes I21-I23; ischemic stroke, ICD-8 codes 433.09, 433.99, 436.01, 436.09, 436.90 or 436.99 or ICD-10 code I63; ischemic heart disease, ICD-8 codes 411-414 or ICD-10 codes I20, I24 or I25.

‡ All hazard ratios were estimated with the baseline hazards stratified by parental birth year (five-year intervals), parental sex, number of daughters with pregnancies in the study period, and total number of children.

**eTable 5. Hazard ratios for any cardiovascular disease by attained age and number of daughters with preeclampsia in parents of daughters with one or more pregnancies of at least 20 weeks’ duration in Denmark, 1978-2017, stratified by daughters’ parity**

| **Parity of daughter** | **Age < 55 years** | | | | | **Age ≥55 years** | | | | |
| --- | --- | --- | --- | --- | --- | --- | --- | --- | --- | --- |
|  | **No. of daughters with PE** | **No. of events**† | **No. person-years (x10^3^)** | **HR**‡ | **95% CI** | **No. daughters with PE** | **No. events**† | **No. person-years (x10^3^)** | **HR**‡ | **95% CI** |
| Nulliparous | 0 | 12,689 | 2,415.5 | 1 | Ref | 0 | 73,963 | 4,986.0 | 1 | Ref |
|  | 1 | 785 | 121.0 | 1.23 | 1.14-1.32 | 1 | 4,567 | 280.0 | 1.13 | 1.10-1.16 |
|  | ≥2 | 14 | 1.4 | 1.77 | 1.05-2.99 | ≥2 | 100 | 5.6 | 1.25 | 1.03-1.52 |
|  |  |  |  |  |  |  |  |  |  |  |
| Multiparous | 0 | 9,657 | 1,526.4 | 1 | Ref | 0 | 157,581 | 10,085.1 | 1 | Ref |
|  | 1 | 766 | 102.4 | 1.18 | 1.09-1.27 | 1 | 12,388 | 707.4 | 1.13 | 1.11-1.15 |
|  | ≥2 | 29 | 2.0 | 2.21 | 1.53-3.17 | ≥2 | 397 | 19.9 | 1.27 | 1.15-1.40 |

CI, confidence interval. HR, hazard ratio. PE, preeclampsia.

† Any cardiovascular disease including: Myocardial infarction, ICD-8 code 410 or ICD-10 codes I21-I23; ischemic stroke, ICD-8 codes 433.09, 433.99, 436.01, 436.09, 436.90 or 436.99 or ICD-10 code I63; ischemic heart disease, ICD-8 codes 411-414 or ICD-10 codes I20, I24 or I25.

‡ All hazard ratios were estimated with the baseline hazards stratified by parental birth year (five-year intervals), parental sex, number of daughters with pregnancies in the study period, and total number of children.

For each attained age group (<55 years and ≥55 years), the associations between preeclampsia in daughters and cardiovascular disease in parents did not differ according to daughters’ parity (nulliparous vs multiparous) (P for interaction=0.59, using Wald Chi square test).

**eTable 6. Hazard ratios for any cardiovascular disease by attained age in parents of daughters with one or more pregnancies of at least 20 weeks’ duration in Denmark, 1978-2017 when the analysis was restricted to the first pregnancy in the first daughter contributing a pregnancy during the study period, with additional adjustment for the daughter’s age at delivery**

|  | **Age <55 years** | | | | | | | **Age ≥55 years** | | | | | |
| --- | --- | --- | --- | --- | --- | --- | --- | --- | --- | --- | --- | --- | --- |
|  | |  |  | **Standard adjustments**‡ | | **Additionally adjusted for daughter’s age at delivery** | |  |  | **Standard adjustments**‡ | | **Additionally adjusted for daughter’s age at delivery** | |
| **No. daughters with PE** | | **No. events**† | **No. person-years (x10^3^)** | **HR** | **95% CI** | **HR*** | **95% CI** | **No. events**† | **No. person-years (x10^3^)** | **HR*** | **95% CI** | **HR** | **95% CI** |
| 0 | | 10,920 | 2,137.7 | 1 | Ref | 1 | Ref | 59,453 | 3,988.6 | 1 | Ref | 1 | Ref |
| 1 | | 565 | 94.5 | 1.17 | 1.08-1.28 | 1.18 | 1.08-1.28 | 2,979 | 178.3 | 1.16 | 1.12-1.21 | 1.17 | 1.13-1.22 |

CI, confidence interval. HR, hazard ratio. PE, preeclampsia.

*Estimates adjusted for maternal age at delivery

† Any ischemic event including: Myocardial infarction, ICD-8 code 410 or ICD-10 codes I21-I23; ischemic stroke, ICD-8 codes 433.09, 433.99, 436.01, 436.09, 436.90 or 436.99 or ICD-10 code I63; ischemic heart disease, ICD-8 codes 411-414 or ICD-10 codes I20, I24 or I25.

‡ All hazard ratios were estimated with the baseline hazards stratified by parental birth year (five-year intervals), parental sex, number of daughters with pregnancies in the study period, and total number of children.

1. **eREFERENCES**

1 Pedersen CB. The Danish Civil Registration System. *Scand J Public Health.* 2011;Jul;39(7 Suppl):22-5.

2 Schmidt M, Pedersen L, Toft Sørensen H. The Danish Civil Registration System as a tool in epidemiology. *Eur J Epidemiol.* 2014;Aug;29(8):541-9.

3 Lynge E, Sandegaard JL, Rebolj M. The Danish National Patient Register. *Scand J Public Health.* 2011;Jul;39(7 Suppl):30-3.

4 Helweg-Larsen K. The Danish Register of Causes of Death. *Scand J Public Health.* 2011;Jul;39(7 Suppl):26-9.

5 Erlangsen A, Feyszyn I. Danish nationwide registers for public health and health-related research. *Scand J Public Health.* 2015;Jun;43(4):333-9.

6 Knudsen LB, Olsen J. The Danish Medical Birth Registry. *Dan Med Bull.*1998;Jun;45(3):320-3.

7 Hypertension G. ACOG Practice Bulletin: Gestational Hypertension & Preeclampsia. *Am Coll Obstet Gynecol*. 2019 Jan;133(1):1.

8 Brown MA, Magee LA, Kenny LC *et al.* Hypertensive disorders of pregnancy: ISSHP classification, diagnosis, and management recommendations for international practice. *Hypertension*. 2018;Jul;72(1):24-43.

9 Johansen M, Kjærgaard N, Legarth J, Salvig JD. Hypertension og præeklampsi. *DSOG Guidel.* 2012;1–32.
